# In Situ Coupling of Ultrasound to Electro- and Photo-Deposition Methods for Materials Synthesis

**DOI:** 10.3390/molecules22020216

**Published:** 2017-01-31

**Authors:** Agnieszka Magdziarz, Juan C. Colmenares

**Affiliations:** Institute of Physical Chemistry, Polish Academy of Sciences, Kasprzaka 44/52, 01-224 Warsaw, Poland

**Keywords:** ultrasound, sonophotodeposition, sonoelectrodeposition, sonophotochemistry, sonoelectrochemistry, cavitation, current density

## Abstract

This short review provides the current state-of-the-art of in situ coupling of ultrasound to chemical deposition methods. Synergetic action of ultrasound and light radiation or electrical fields may result in new powerful methodologies, which include sonophotodeposition and sonoelectrodeposition processes. The effect of ultrasound is explained on the basis of different physical mechanisms emerging from cavitation phenomenon. Some possible mechanisms of the interactions between ultrasound and photochemical and electrochemical processes are discussed here. The application of sonophotodeposition and sonoelectrodeposition as green energy sources in the syntheses of different nanomaterials is also reviewed.

## 1. Introduction

The fast development of sonochemistry started around a few decades ago when cheap ultrasonic equipment, such as the ultrasonic bath, started to be a common equipment in many chemical laboratories. This was also when the coupling of ultrasound with other chemical methods, such as electrochemistry or photochemistry began to be widely studied [1]. In his works Compton claimed that combination of two independent sources of energy often results in formation of new powerful methodologies [2,3]. In the mentioned processes, electrical and mechanical green energies are delivered to the reaction systems. In this way a high-energy microenvironment is created, that enables many chemical reactions to proceed thereby eliminating the necessity of the use of harmful and dangerous reagents and solvents [4]. Also, some operational variables (e.g., ultrasonic power and frequency, electric current) are available in these methods giving the possibility to control the kinetics of the process by their proper adjustment [4]. It is commonly known, that ultrasonic radiation (from 20 kHz to 1 GHz) is too weak for rupturing chemical bonds and that the acoustic filed cannot couple directly with molecular energy levels [5,6]. However, the extreme conditions (very high temperature and pressure), that are formed during the expansion of ultrasound in the liquid, are responsible for breaking the chemical bonds. This cavitation phenomenon together with other related mechanical phenomena such as acoustic streaming and microjetting, shockwaves, promotion of mass transport, as well as chemical effects can influence the electrochemical and photochemical processes [7].

The application of sonoelectrochemistry as well as sonophotochemistry is a very broad topic in chemistry. Therefore, in this short review we will focus on a very narrow combination of sonication with electrochemical deposition and sonication with photochemical deposition. Moreover, only their simultaneous use will be analyzed (in situ coupling). In the next part the interaction between ultrasound activation in the liquid environment and electrochemical and photochemical reactions will be explored. In the last part of this review some examples of the various materials prepared by sonophoto-and sonoelectrodeposition methods are described paying particular attention to their morphological properties, chemical composition, and the influence of ultrasound during the synthesis procedure.

## 2. Mechanism of Sonoelectrodeposition and Sonophotodeposition Processes

**Figure 1:**
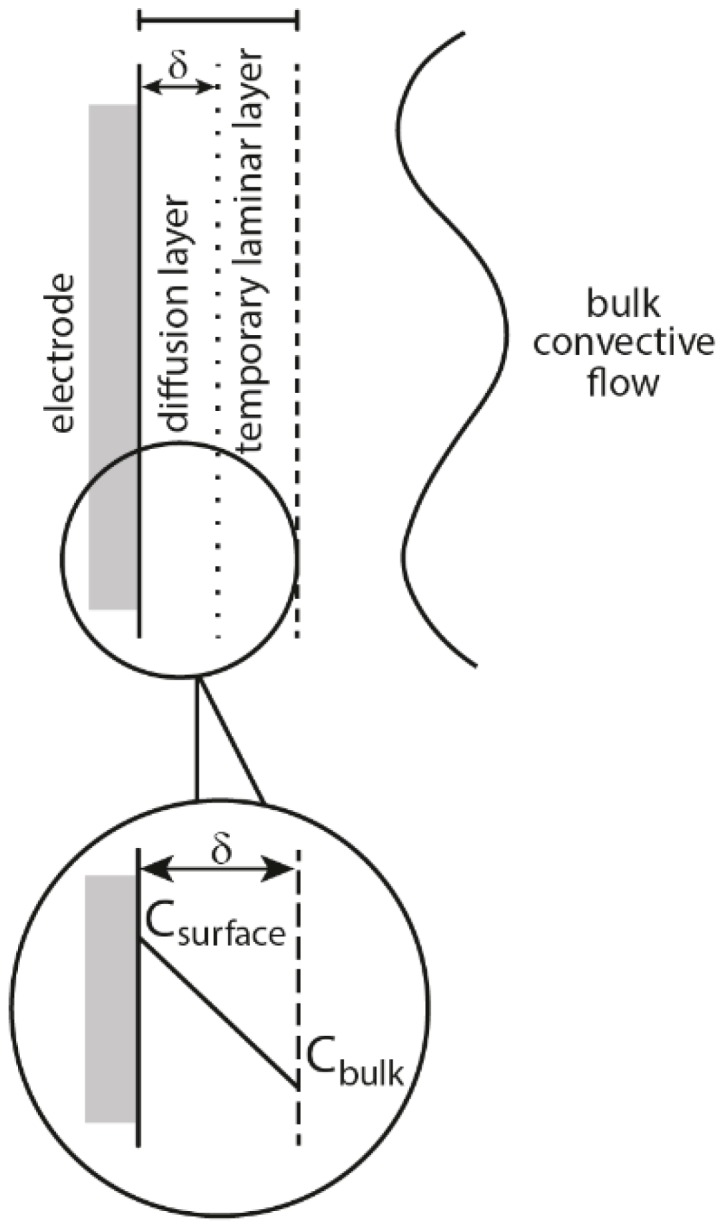
Schematic representation of the diffusion and boundary layers at the electrode-solution interface as applied in the diffusion layer model. Reprinted from [20] by permission of John Wiley & Sons, Inc.

According to J. Klima there are four possible mechanisms of interaction between sonication and electrochemical processes equivalent to physical effects of ultrasound in liquids: (i) acoustic streaming, (ii) microstreaming and turbulence, (iii) microjets and (iv) shock waves [1]. Acoustic streaming is created when ultrasound passes through a liquid and a part of its energy is absorbed, which can be observed as a movement of the solution. It can enhance the mass transfer of the electroactive compounds, which is seen as an increase of electrochemical current and in the change of the shape of voltammetric curves [8,9]. Interestingly, an increase in current is not only observed in the place where ultrasonic energy is absorbed, but also can be seen if the working electrode is located outside of this region. This is caused by the radiation pressure, that can move the flow of liquid away from the absorption center. During turbulence, an increase of electrochemical current, similar to that obtained in acoustic streaming occurs, but only when the working electrode is placed in the region of turbulent motion. This effect results from the intense cavitation caused by mutual interaction of oscillating and collapsing bubbles. Microjets are formed as a consequence of the collapse of cavitational bubbles at or near the solid-liquid interface and are directed towards the electrode surface [10]. Their formation is a result of an intense transient cavitation in the vicinity of the electrode and can be seen as current pulses. Shock waves appear under some conditions after the violent bubble collapse and can erode the surface of the electrode. As a result, a new surface is formed, which is seen in the form of increased current pulses. This process occurs only with cavitation in close vicinity of the electrode surface. The main effect of physical activity of ultrasound on the electrochemical process is the enhanced mass transfer, which is manifested by an increase of electrolytic current. However, this increase of current depends on the applied mechanism; turbulence, microjets, and shock waves derived from cavitation taking place near the working electrode. Thus, under some conditions these three effects can appear together decreasing significantly the diffusion layer (far under 1µm), which is schematically presented in Figure 1. In comparison, with the acoustic streaming the diffusion layer thickness can be reduced only to several micrometers, which means also a smaller increase of current. Among the mentioned mechanisms microjets are the most efficient in reduction of the diffusion layer and show the widest spectrum of use in electrochemistry. Apart from enhanced mass transfer of substrates and products to and from the electrode, they can also remove the absorbed material or even passivate the layer at the electrode [11,12,13]. This is enabled by the formation of very high pressure (several thousand bars) on the electrode surface at the moment of the jets of liquid reaching the surface [14]. On one hand, the activation of the electrode can occur, but on the other also its corrosion [15]. Therefore, cavitation can produce two opposite effects: mass transfer enhancement, which facilitates film formation, and reactivation of the electrode, which means destruction of the film layer. Apart from the influence of physical effects of ultrasound on the mechanism of electrodeposition, chemical effects also can play an important role. According to Garcias’ group the formation of OH^•^ radicals in the process of sonolysis enhances the formation of nucleation centers by their adsorption on the electrode surface [16,17,18]. An interesting result of the study of nucleation mechanism in the sonoelectrodeposition process can be found in the work of Mallik et al. [19]. It reveals, that ultrasound is capable of breaking crystal bonds of the primary nuclei and on the defect-free crystal face induces secondary nucleation. Of course this process cannot occur infinitely long, due to increased adherence under sonication. The deposits obtained in this process consisted of reduced grain size in contrast to coarse grain deposits produced without sonication.

Increased hardness of the deposit, enlarged film thickness, improved deposition rates and efficiencies, greater adhesion of the deposits to electrode, and brightness enhancement are the most often mentioned benefits of electrodeposition of metals assisted with ultrasound [21,22,23]. The brightness of deposits depends on the current density and it decreases when current density increases above a critical value. The use of ultrasound increases the critical current density and helps to obtain electrodeposits with increased brightness. The ultrasound effects that influence the brightness come from shock waves followed by cavitation erosion. As a result, films built of fine grain size and higher grain-packing density are produced.

**Figure 2:**
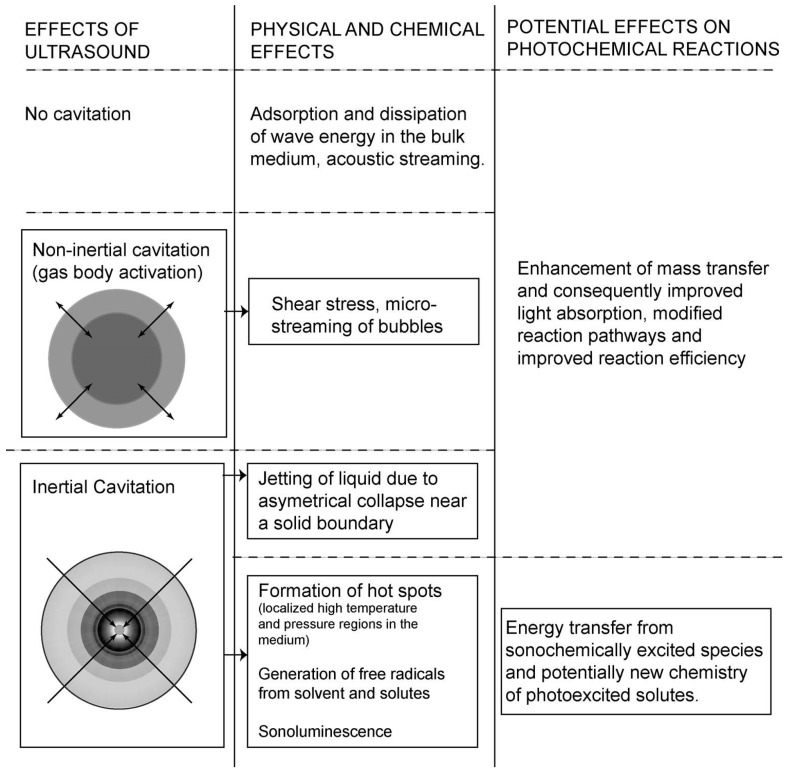
Interaction of ultrasound effect with photochemical reactions. Reprinted from [6] with permission of Springer.

**Figure 3:**
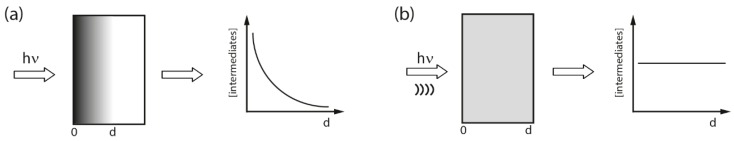
Distribution of excited states in the photochemical process (**a**) and after addition of sonication (**b**). (Dots represent the density of activated species). Adapted from [24] with permission from Elsevier.

The synergism between sound and light in sonophotochemical processes is most often correlated with the enhanced mass transfer induced by ultrasound that can affect the photochemical reaction rates [6]. However, the interaction between sono- and photochemical irradiation strictly depends on the type of ultrasound effect produced in the liquid, which is shown in detail in Figure 2. It can be seen that this enhanced mass transfer can in consequence improve light absorption and modify the reaction pathway. The energy transfer to molecules occurs only when cavitation is produced. The transfer of photoexcited states in a pure photochemical system is not homogeneous (see Figure 3a), because it depends on how light propagates through an absorbing medium and normally its intensity decreases with a distance [24]. It is also known, that the reaction rate in photochemical process depends on the concentration of the excited states. Figure 3b predicts how the distribution of a photoexcited stated could change when sonication is added to the system. An intense mixing provoked by cavitation and shock waves should distribute the excited states in the whole reaction volume causing the reaction rates to be more or less the same at each point of the solution. However, not only cavitation is the important factor, but also the relative formation rates, lifetimes, and further decomposition of excited species. Additionally, it was suggested that long-lived excited states could be quenched by cavitation. Another aspect of “sonophoto” synergy can be found in some works where it is said that the combined effects of ultrasound and light could potentially generate a new chemistry of photoexcited solutes under the extreme conditions inside cavitation microreactors [6]. The Colmenares group performed an experiment comparing the deposition of iron on TiO_2_ semiconductor, in order to obtain iron oxide, using ultrasound, light, and ultrasound coupled with light (results not published). They compared the morphology and chemical structure of these three samples to find out if a new chemistry occurred. They discovered that nanostructured hematite was the main form obtained in the sonophotodeposition as well as in the photodeposition process. In contrast, different iron oxide forms were registered after sonodeposition. This could be interpreted in that the same mechanism, mainly photocatalytically-induced electrons is responsible for the reduction of the iron precursor after compilation of “sono” with “photo”. On the other hand, a clear physical effect of ultrasound was seen in a better dispersion of Fe_2_O_3_, which was localized not only on the surface, but also in the bulk of the material. An important issue in this experiment is the reaction solution consisting of 30% of water and 70% of acetonitrile. In such environment the reductive power of sonication was probably reduced, because only a low amount of H^•^ radicals with high reductive power could be formed. As a result, a mechanical role of sonication and a chemical role of light were found in the sonophotodeposition process in this particular case. 

## 3. Materials Obtained by the Sonoelectrodeposition and Sonophotodeposition Processes

**Figure 4:**
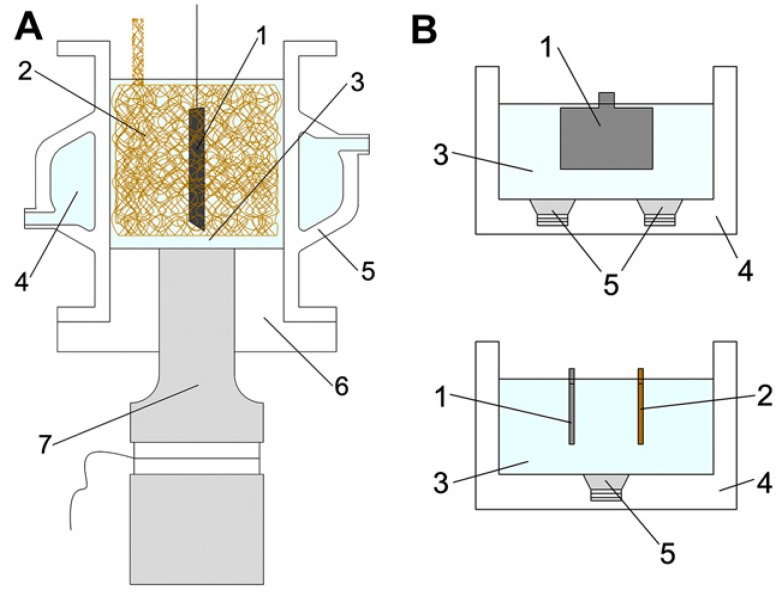
(**A**) 20 kHz sonoreactor adapted as a sonoelectrochemical device: (1) lead dioxide electrode, (2) copper counter-electrode (foam geometry), (3) working solution, (4) cooling jacket, (5) glass cell, (6) Teflon holder, (7) ultrasonic transducer. (**B**) 40 kHz ultrasound bath: (1) lead dioxide electrode, (2) copper counter-electrode, (3) working solution, (4) ultrasonic bath, (5) ultrasonic transducers. Reprinted from [25] with permission from Elsevier.

In this part we discuss the examples of the materials prepared by using sonoelectro- and sonophotodeposition methods, see also Table 1. The sonoelectrodeposition has already been used for preparation of vast number of materials. Herein, we describe several examples showing the diversity of the materials that can be obtained by using this method. Sonoelectrodeposition of lead oxide on glassy carbon and platinum electrodes was studied by Garcia et al. [16] (Table 1, Entry 1). In this experiment a commercial ultrasonic bath (30 kHz, 100 W) together with a platinum electrode and a glassy carbon rod as working electrodes were used. The authors observed that the behavior of these electrodes under action of ultrasound was different. The response of the platinum electrode was unchanged in comparison with silent conditions. In contrast, activation of the glassy carbon electrode during electrodeposition of lead dioxide was observed when the ultrasound was applied. Interestingly, no changes were observed in the surface topography of the electrode. Therefore the activation of the electrode was directly correlated with the formation of some surface functional groups in the reaction of OH^•^ radicals, formed in the aqueous sonolysis, with the carbon surface. These groups play the role of intermediates in the mechanism of lead oxide deposition and are consumed as the reaction progresses. The influence of ultrasound power and frequency on sonoelectrodeposition of PbO_2_ on a glassy carbon electrode was studied in the subsequent works of Garcia’s group [17,18] (Table 1, Entry 2). The sonoelectrochemical reactor consisting of a jacketed Sonoreactor (20 kHz, 100 W max. power) was used in these studies. The authors found that the increase of power as well as frequency of ultrasound had an influence on the kinetics of the electrodeposition process, increasing the nucleation constant and decreasing the induction time. This was correlated with the enhanced generation of OH^•^ radicals, which can be adsorbed on the electrode surface and therefore the number of nucleation centers should be increased. A formed nucleus favors the lead oxide deposition. However, the potential of the electrode also influences the electrodeposition, therefore these experiments were performed under conditions where the electrodeposition process was not favored. Glassy carbon was also used as a substrate in the next experiment with deposition of lead oxide film performed by Saez et al. [25] (Table 1, Entry 3). They used two experimental setups, one consisted of a sonoreactor (20 kHz) and plates of graphite as substrate electrodes and in the other one an ultrasound bath (40 kHz, 100 W) with larger plates of graphite or titanium as electrodes were applied (see Figure 4). The obtained results were compared with the mechanical agitation procedure. The authors discovered that only the ultrasound field enhanced the electrodeposition of lead oxide providing a finer grain, more homogeneous, stress-free and nodule-free film deposit on both substrates. Acoustic streaming and microjets were mainly responsible for the good adherence of the film on the coarse and rough surface of the electrode substrate. The only drawback was a random presence of pores. To avoid them, the specific pretreatment of the electrode surface was suggested as well as study of the application of high-frequency ultrasound fields.

The first trial of sonoelectrodeposition of platinum on a glassy carbon electrode was reported by Pollet et al. [26] (Table 1, Entry 4). For this, a special jacketed cooling electrochemical cell with a 20 kHz ultrasonic probe was designed. It was confirmed by the authors that for the formation of Pt deposits a substantial overpotential is required and that the whole process is irreversible. In the process driven under sonication the positive shift in potentials provoked a decrease in concentration and nucleation overpotentials thus facilitating the electrodeposition of platinum. They also observed a significant difference between morphology of the Pt deposits obtained under silent conditions in comparison with forced convection conditions, like rotation and sonication. Individual Pt nanoparticles deposits were produced with no agitation whereas agglomerates of larger Pt nanoparticles were formed under sonication and rotation. In this work, the use of continuous ultrasound had a detrimental effect on the electrocatalytic performance of the fuel cell in comparison with the pulsed ultrasound used in galvanostatic deposition reported earlier by the same author [27]. Later on, more sophisticated platinum structures were obtained by using sonoelectrodeposition. Heli et al. [28] reported the synthesis of platinum hierarchical nanoflowers, whereas fractal nanoplatinum structures were synthesized by Taguchi et al. [29]. More information on these materials is collected in Table 1, Entries 5 and 6, respectively.

**Figure 5:**
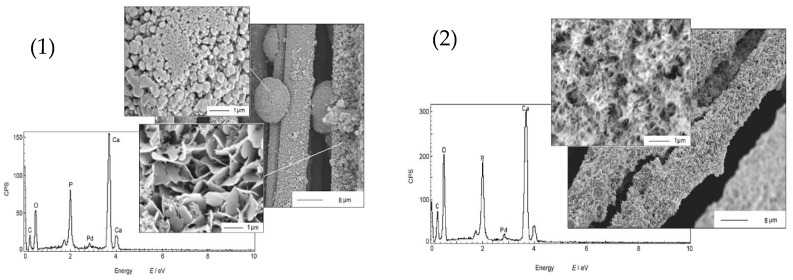
Morphologies and composition of CaP obtained by: (1) electrodeposition in the absence of sonication vs. (2) sonoelectrodeposition. Reprinted from [30] with permission from Elsevier.

Biomedical materials were synthesized using sonoelectrochemical deposition by Han et al. [30] (Table 1, Entry 7). Calcium phosphate (CaP) coatings were deposited on carbon fibers creating bioceramic composites which could be used to reconstruct bone defects. For this purpose, highly irregular objects often need to be coated and the electrodeposition method makes this task easier in comparison with other available methods. The synthesis setup consisted of an ultrasonic bath (40 kHz, 2.16 W/cm^2^), carbon fabric as the working electrode and the whole procedure was performed under a constant current density (20 mA/cm^2^). The authors observed that the action of ultrasound significantly influences morphology as well as the chemical composition of the CaP coatings. Figure 5 compares the morphology of the coatings electrodeposited under silent mode vs. sonoelectrodeposited. It is clearly seen that application of silent mode resulted in the formation of a porous layer with plate-like crystals (~1 µm) or small spheres. In contrast, very uniform coatings, with small needle-like crystals (<0.1 µm) and a good adhesive strength were obtained under sonication mode. Regarding the chemical structure it was discovered that application of ultrasound promoted the formation of PO_4_^3−^ instead of HPO_4_^2−^. Concluding, the CaP coatings obtained with the use of ultrasound were more accepted by the osteoblast cells.

**Figure 6:**
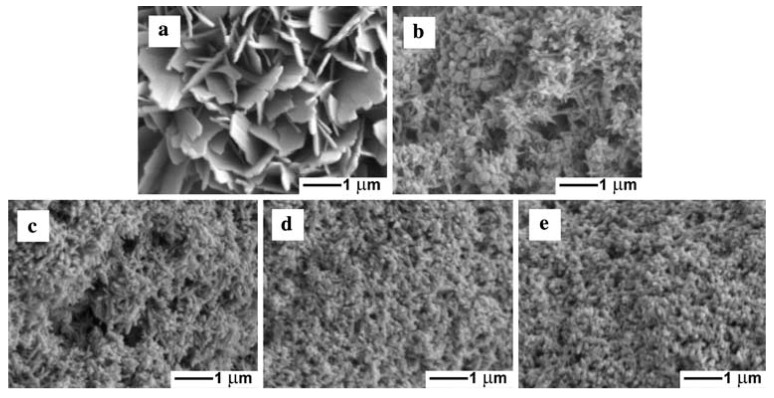
Scanning electron microscopy (SEM) micrographs of the samples prepared by sonoelectrodeposition under different current densities. (**a**) 5 mA/cm^2^; (**b**) 8 mA/cm^2^; (**c**) 13 mA/cm^2^; (**d**) 20 mA/cm^2^; and (**e**) 34 mA/cm^2^. Reprinted from [31] with permission of Springer.

The influence of different current densities (5, 8, 13, 20, and 34 mA/cm^2^) on morphologies and structures of CaP coatings on carbon surfaces prepared by sonoelectrodeposition was studied by Han et al. in the next work [31] (Table 1, Entry 8). It was observed that morphology of the CaP deposits changed with increasing current density (see Figure 6). The plate-like crystals were formed at the lowest current density 5 mA/cm^2^, a mixture of spherical and needle-like structures appeared at 8 mA/cm^2^. The further increase of the value of current density resulted in the formation of needle-like morphology with more uniform structures and smaller crystal sizes as the current density grew. It was concluded that at the lower current density an insufficient concentration of OH^−^ ions was the cause of formation of octacalcium phosphate. On the other hand, the higher current densities and the needle-like morphology confirmed the formation of the apatite form of calcium phosphate.

Another work in this field was dedicated to the formation of hydroxyapatite/porous carbon composite scaffolds and reported by Liu et al. [32] (Table 1, Entry 9). The obtained material was homogeneously coated with the needle- or sheet-shaped crystals of hydroxyapatite such as on the external and internal surface of the scaffold. This uniform coating ensured an adequate microenvironment and promoted the adhesion and proliferation of osteoblast cells, no matter of the location on the porous carbon scaffold. The biomedical application of CaP coatings makes them an interesting topic for many research groups and therefore, there are many works available in the literature. Also Zhao et al. obtained a nano/micro-sized CaP coating prepared on carbon/carbon composites [33] (Table 1, Entry 10). The obtained coating consisted of hydroxyapatite and brushite. The authors observed that at different deposition voltages the morphology of the crystals changed. In the most homogenous coating (voltage of deposition 2.4 V) the nano-sized and needle-like crystals of hydroxyapatite were embedded in a micro-sized and plate-like structure of brushite. This homogeneous structure resulted in an enhanced adhesive and cohesive strength of the coating.

**Figure 7:**
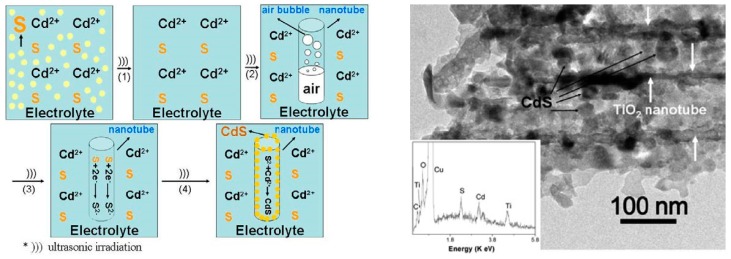
On the left: a schematic diagram of the possible sonoelectrochemical deposition interaction. On the right: a transmission electron microscopy (TEM) image of CdS-TiO_2_NTs after CdS sonoelectrochemical deposition with the inset energy dispersive X-ray (EDX) spectrum. Reprinted from [34]. © IOP Publishing. Reproduced with permission. All rights reserved.

An intriguing work was performed by Wang et al., who filled in TiO_2_ nanotubes with CdS nanoparticles through a single-step sonoelectrodeposition method [34] (Table 1, Entry 11). In this experiment TiO_2_NTs as a working electrode, and an ultrasonic bath (40 kHz, 2.4 kW/m^2^) under constant current density (5 mA/cm^2^) were used. Deposition of CdS nanoparticles inside the TiO_2_ nanotubes was facilitated by the action of ultrasound, which is shown in Figure 7. First of all, ultrasound promoted dissolution of elemental sulfur. Next, it enhanced expelling of the air from the nanotubes, which was a process that enabled the ions to enter inside the tube. Finally, ultrasound helped to obtain smaller and uniformly sized CdS nanoparticles. A real effect of this process is presented below in the transmission electron microscopy (TEM) image (Figure 7, on the right). An ordered tubular structure of TiO_2_ with CdS nanoparticles deposited into the channels is clearly seen. This result also confirms that ultrasound did not damage the ordered structure of the TiO_2_NTs during sonoelectrodeposition of CdS nanoparticles. Such obtained CdS-TiO_2_NTs electrodes gave a stronger photocurrent and an extended photoresponse under visible light (up to 480 nm) in comparison with the CdS-TiO_2_NTs electrodes prepared using electrodeposition without sonication.

**Figure 8:**
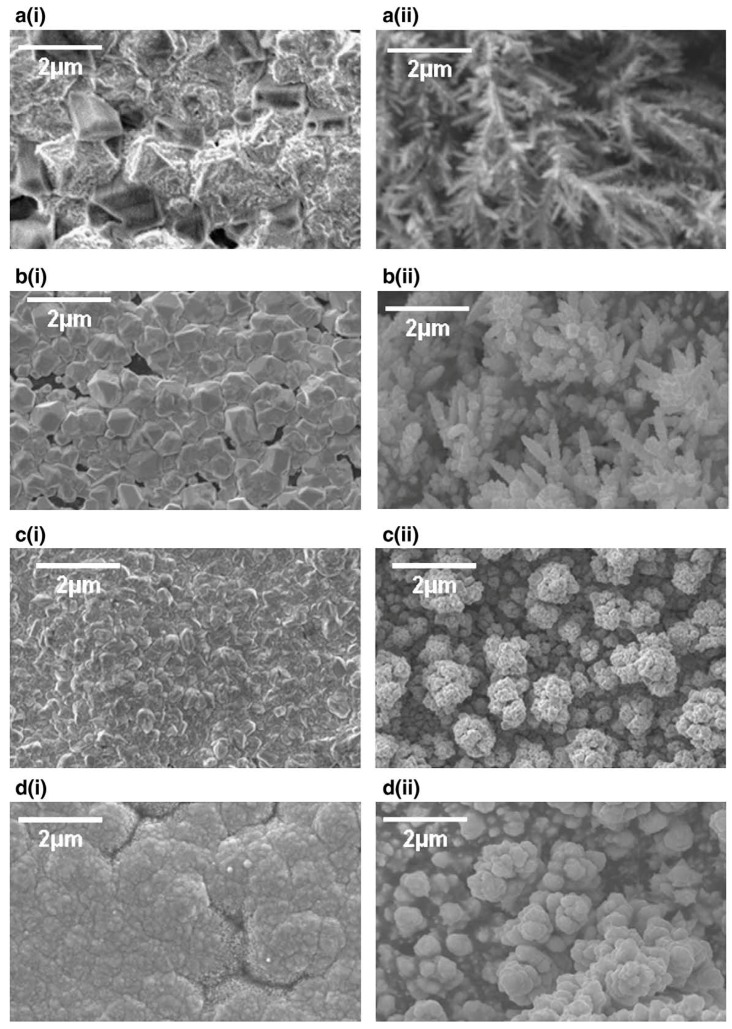
SEM images of copper deposits with (i) and without (ii) sonication at (**a**) 25, (**b**) 19.5, (**c**) −1 and (**d**) −3 °C. Reprinted from [36] with permission from Elsevier.

The impact of deposition temperature on the residual stress of Cu films sonoelectrodeposited on the graphite substrate was investigated by Mallik et al. [35] (Table 1, Entry 12). The experiments were carried out on a graphite electrode using an ultrasonic probe (20 kHz, 20% output) at different temperatures: 25, 20, 15, 10, and 5 °C. It was observed that the type of residual stress in the Cu films was only compressive, which means that films “want” to expand, at each applied deposition temperature. The maximum value of compressive residual stress was registered at the lowest deposition temperature, 5 °C. The mechanical properties of the films are strongly related with the residual stress. Therefore, the maximum values of hardness, elasticity, and surface adhesion at the grain surface were also obtained at 5 °C. This could be correlated with the effects of residual compressive stresses on suppressing crack propagation. Interestingly, an opposite tendency was observed for adhesion at grain boundaries, which decreased together with the deposition temperature. Such behavior was ascribed to changes in energy of the grains on the surface and at the boundaries as well as to some interfacial tension occurring during the atomic force microscopy (AFM) measurement. Ultrasound was reported to have a significant enhanced effect on the magnitude of the compressive stress by increasing the adatom mobility and attachment of the islands with a substrate. Also the impact of low deposition temperature on the morphology of the copper deposition was studied by Mallik et al. [36] (Table 1, Entry 13). Temperatures selected for this experiment were as following: 25, 19.5, −1 and −3 °C, and an ultrasonic bath (30 kHz, 60 W) was the source of sonication. Different results between sonicated and silent conditions as well as between ambient and low temperatures are clearly seen in Figure 8. Inhomogeneous morphology consisted of near granular as well as highly distorted areas with some perforated grains obtained at 25 °C under sonication (Figure 8a (i)). This distortion was explained by the ablating effect of microstream jetting. At lower temperature 19.5 °C and under sonication (Figure 8b (i)) bimodal faceted Cu structures with bigger and smaller grains were deposited. Enhanced mass transport by ultrasound, improved bulk deposition, simultaneous nucleation, and grain growth were some of the factors responsible for this result according to the authors. The corresponding samples at silent conditions (Figure 8a (ii) and Figure 8b (ii)) presented dendritic structures. Totally different morphology of Cu films was obtained at −1 °C temperature with sonication. In Figure 8c (i) a uniform copper covered surface with well agglomerated spheroids is seen. The low temperature, favoring the formation of small nuclei, improved the rate of mass transfer, creating enhanced nucleation, as well as degassing of the electrode surface by ultrasound, giving adherent and bright deposits, which were favorable for the obtained morphology. Similarly, the deposit formed at −3 °C presented a uniform and fully coalesced fine grained Cu coating (Figure 8d (i)). Conversely, the deposits obtained at silent mode (Figure 8c (ii) and Figure 8d (ii)) showed branched, not dense structures.

**Figure 9:**
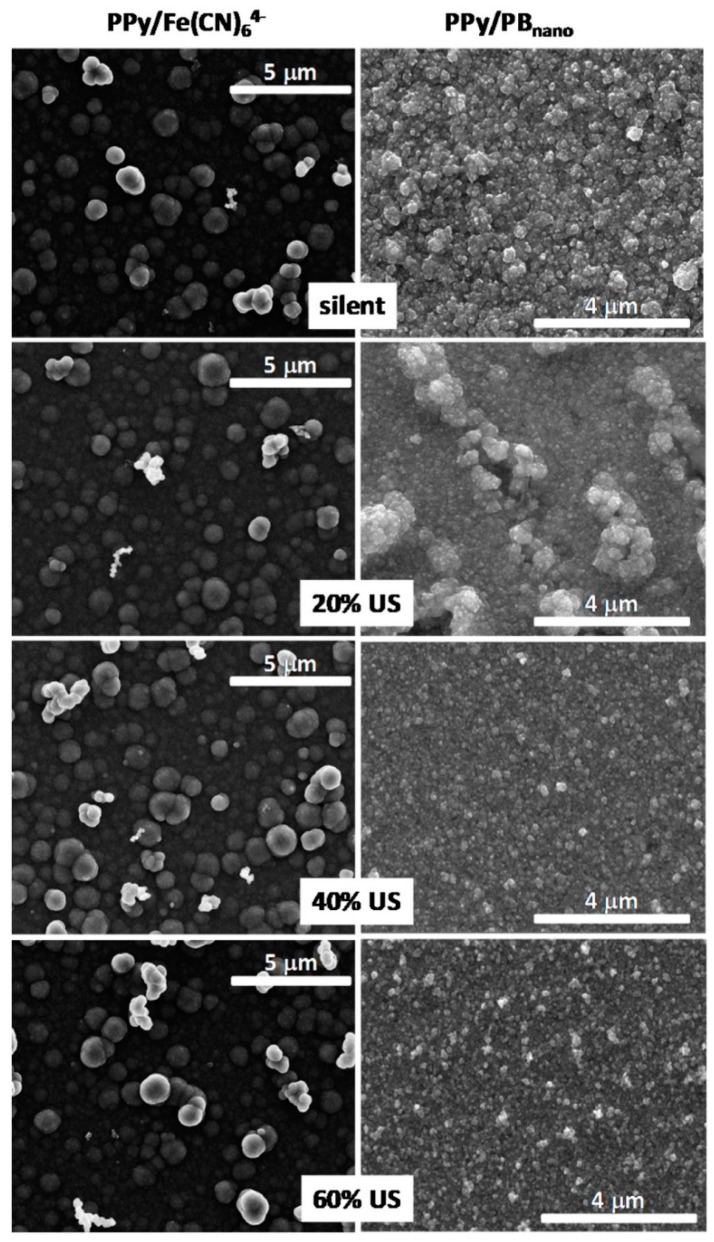
Representative FEG-SEM images taken from ITO electrodes modified by PPy/Fe(CN)_6_^4−^ and PPy/PB_nano_ employing different US amplitude. All electrodes were modified by the same PPy deposition charge 160 mC/cm^2^. Reprinted from [37] with permission from Elsevier.

The effect of ultrasound amplitude on the formation of poly(pyrrole) PPy/Prussian blue (PB) and poly(pyrrole) Ppy/dodecylbenzenosulphonate (DBS) nanocomposites was investigated by Hostert et al. [37,38] (Table 1, Entries 14 and 15). The following amplitudes of ultrasonic horn (20 kHz, 130 W) were checked at: 20%, 40%, and 60%. It was found that by changing the amplitude of ultrasound the morphology and distribution of nanoparticles on the electrode can be tuned. The influence of different US amplitude on the ITO electrode modified by PPy/Fe(CN)_6_^4−^ and PPy/PB_nano_ is presented in Figure 9 [37]. A similar globular morphology is clearly seen under silent as well as at each US amplitude for the control electrode non-modified with Prussian blue. In contrast, on the surface of PPy/PB_nano_ electrode the globules with inorganic structures are only seen at 20% amplitude conditions. At the higher amplitudes (40% and 60%) rather smooth surfaces with very small aggregates are seen, which can originate both organically and inorganically. This enhanced surface morphology was explained by better inclusion of small PB_nano_ nanoparticles within PPy film under increased sonication. The PPy/PB_nano_ electrode presented enhanced electrochemical features such as good stability over continuous cycling and sensitivity towards reduction of H_2_O_2_. Similarly, a smoother film deposit with a distinct voltammetric behavior was obtained in the case of Ppy/DBS electrodes under sonochemical conditions [38]. It was also observed that the use of ultrasound facilitated the charge-transfer process at the Ppy/electrolyte interface and resulted in different charge distribution, explained by different amounts of intercalated DBS anions.

**Figure 10:**
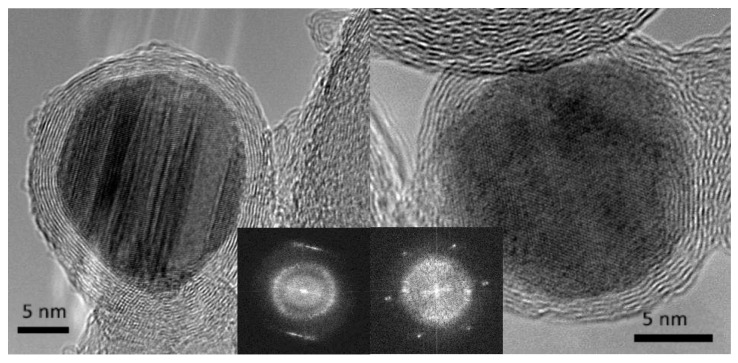
HRTEM images of typical encapsulated nanoparticles, with (inset) the central portions of their respective Fourier transforms to indicate the good crystallinity of both core (sharp spots/streaks in the Fourier transform) and the well-defined ring that arises from the carbon shell. Reprinted from [39] © IOP Publishing. Reproduced with permission. All rights reserved.

A series of work regarding formation of bimetallic nanoparticles by using sonoelectrodeposition method was reported by Luong et al. [39,40,41]. Co-Pt nanoparticles were sonoelectrodeposited and then thermally treated under CO to become encapsulated within carbon cages [39] (Table 1, Entry 16). For this aim an ultrasonic horn was used in a dual role: as a cathode and ultrasound emitter working in a pulse mode. The authors observed the formation of a heterogeneous CoPt film with separate population of Pt-rich and Co-rich particles after the sonoelectrodeposition process. This was explained by different deposition rates of these elements, because of the differences in their standard electrode potentials. Annealing in the CO resulted in the creation of many core-shell structures with metallic cores and carbon-onion shells (see Figure 10). Also a hard magnetic Co-Pt phase was registered. A similar reaction setup was used for the preparation of FePt nanoparticles [41] (Table 1, Entry 17), although the annealing step was performed under H_2_. Similarly to the previous example the sonoelectrodeposition of Fe and Pt led to formation of small domains of pure Fe and Pt. Again, a large difference between the standard electrode potential of Fe^2+^ and Pt^4+^ was given as a reason for the lack of alloy formation. After annealing, when diffusion processes occur, the ordered L1_0_ face-centered tetragonal (fct) FePt phase was registered. Also, the saturation magnetization and the coercivity of the nanoparticles were improved after the annealing process. Similar results were obtained in the recent work of this group reporting the synthesis of FePd nanoparticles [40] (Table 1, Entry 18). The annealing process under (Ar + 5% H_2_) atmosphere also resulted in the formation of an ordered L1_0_ fct FePd structure with hard magnetic properties. In the electrodeposition of metallic nanoparticles ultrasound helped to remove the nanoparticles which were growing on the cathode surface.

**Figure 11:**
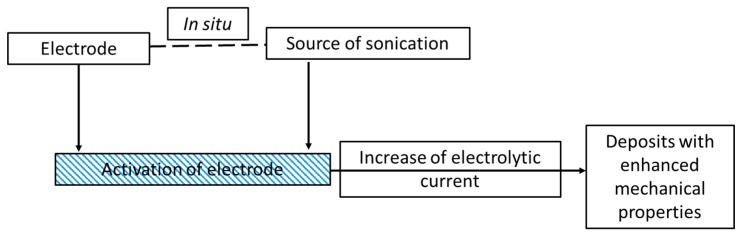
A schematic representation of the synthesis of the materials by means of the sonoelectrodeposition method.

It is worth mentioning other researchers and their contribution to sonoelectrodeposition studies, however we will not discuss them in detail here. Chang et al. reported preparation of Ni-Co/Al_2_O_3_ composite coating with improved properties due to the use of ultrasound [42] (Table 1, Entry 19). Better properties of Zn-Ni-Al_2_O_3_ coating electrodeposited in ultrasound assistance, especially corrosion resistance and hardness, were also found in the works done by Zheng et al. (Table 1, Entries 20 and 21) [43,44]. Noble metals Ag and Au structures were also synthesized by using sonoelectrodeposition. Cheng et al. reported the formation of differentially shaped Ag nanoparticles [45] (Table 1, Entry 22), Tuan et al. obtained Ag NPs loaded on active carbon [46] (Table 1, Entry 23) whereas the synthesis of gold nanorods was proposed by Rahi et al. [47] (Table 1, Entry 24). An interesting combination of sonoelectrodeposition with galvanic replacement was proposed by Rousse et al. [48] (Table 1, Entry 25) for the synthesis of copper-silver bimetallic nanopowders. Sonoelectrodeposition was used for the preparation of the inner copper core whereas the galvanic reaction allowed the replacement of surface copper atoms by silver atoms forming an outer shell. The synthesis of iron-chromium alloy nanoparticles employing pulsed sonoelectrodeposition was performed by Zin et al. [49] (Table 1, Entry 26). The effects of different synthesis parameters, such as bath temperature, pH, and electrolyte composition, on process efficiency and nanoparticles features were studied. The effect of sonication on electroplating of iridium was investigated by Ohsaka et al. [50] (Table 1, Entry 27). A very interesting work comparing direct current electrodeposition and pulse electrodeposition with sono-assisted direct current electrodeposition and sono-assisted pulse electrodeposition in the synthesis of the NiZnS electrode was done by Seetharaman et al. [51] (Table 1, Entry 28). Pulse electrodeposition enabled higher cathodic current density to be applied at the electrode interface and to control surface properties of the composite coatings. Sonication added to this method increased surface smoothness and decreased the residual stresses of the electrode. The best performance in the oxygen evolution studies showed an electrode prepared by the sono-assisted pulse electrodeposition method. A simplified scheme reflecting the key of sonoelectrodeposition is presented in Figure 11.

**Figure 12:**
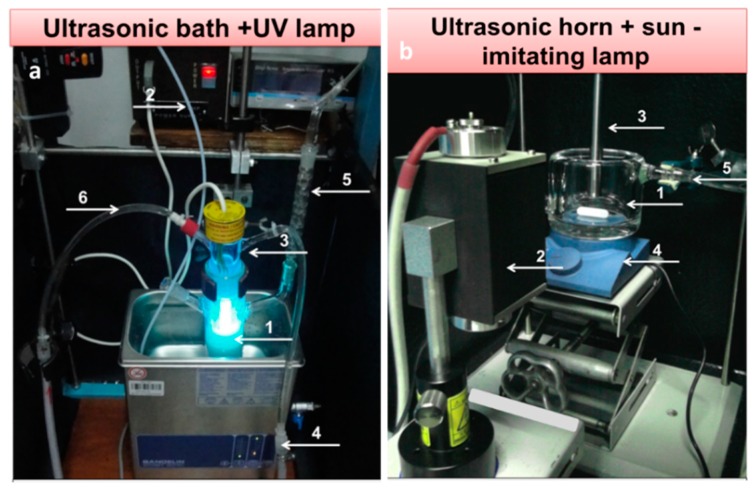
Different reaction setups for the sonophotodeposition synthesis: (**a**) US bath-UV lamp setup: (1) batch photoreactor, (2) argon line, (3) 6 W UV lamp, (4) ultrasonic bath, (5) reflux condenser (6) lamp cooling system; (**b**) US horn-Xe lamp setup: (1) photoreactor, (2) 150 W Xe lamp, (3) ultrasonic horn, (4) magnetic stirrer and (5) cooling system.

**Figure 13:**
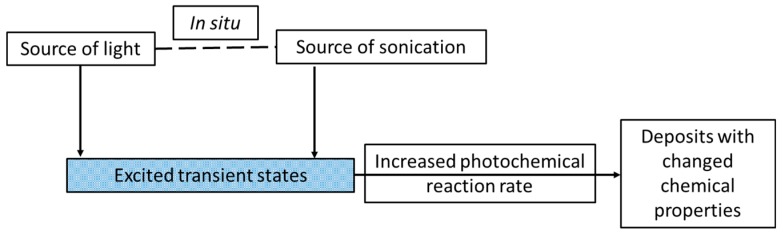
A schematic representation of the synthesis of the materials by means of the sonophotodeposition method.

On the other hand, not many examples concerning materials obtained by the sonophotodeposition method could be found in the literature. For example, this method was implemented and studied by Colmenares group. For the last two years they have reported preparation of several titania-based photocatalytic systems. We will briefly overview these works. The first trial of sonophotodeposition of metal nanoparticles on the surface of a semiconductor concerned palladium on the commercial TiO_2_ P90 photocatalyst [52] (Table 1, Entry 29). The reaction setup consisted of an ultrasonic bath and a 6W Hg lamp (λ_max_ = 254 nm), which is presented in Figure 12a. In all photocatalyst (0.5 wt%, 1 wt%, and 2 wt% Pd) palladium was totally reduced to the metallic form although the air calcination step was the last in the synthesis procedure. These results of sonophotodeposition were compared with the traditional photodeposition where the palladium oxide was the only detected form on the surface. Therefore, the crucial role in formation of Pd° was ascribed in the sonophotodeposition method to sonochemically formed radicals. The authors claimed that these radicals could additionally remove the oxygen atoms from the organometallic structure of the palladium precursor. As a result, the residual carbon combined with the oxygen from the air flow and palladium was not oxidized. The same semiconductor, TiO_2_ P90, and the same reaction setup were then used in the synthesis of bimetallic samples Pd-Au/TiO_2_ and Pd-Cu/TiO_2_ reported in the next two articles [53,54]. The simultaneous sonophotodeposition of palladium and gold resulted in the complete reduction of gold whereas palladium was partially reduced to the metallic form as well as the PdO form being also detected [53] (Table 1, Entry 30). The authors observed the formation of random alloys between the Pd-Au nanoparticles as well as a strong metal-support interaction effect (SMSI) between these nanoparticles and TiO_2_. According to the authors the formation of SMSI effect could be a result of the shock waves and interparticle collisions produced by cavitation. It is important to mention that this effect was observed only in the case of one photocatalyst with a specific composition of metals (1 wt% Pd50-Au50/TiO_2_ P90), which means that the amount of the introduced metals must be optimized. The SMSI effect had a strong influence on the photocatalytic results, in terms of gas phase conversion of methanol and selectivity to methyl formate. In contrast, the monometallic 1 wt% Pd/TiO_2_ P90 photocatalyst was not active in the selective photoreaction towards methyl formate. Similarly, in the sonophotodeposition of palladium and copper, metallic copper and palladium as well as palladium oxide were obtained [54] (Table 1, Entry 31). The partial reduction of palladium was explained by the presence of copper and the retarding effect that it could have on the total reduction process of Pd. Also the SMSI effect was observed for 1 wt% Pd-Cu(1-1)/TiO_2_ P90 photocatalyst, which at the same time was the best performing material in the gas phase oxidation of methanol. Concluding, the addition of a second metal, gold or copper, to palladium significantly changed the reaction route. Generally worse results in this reaction were obtained for the photocatalysts prepared by the photodeposition method. In the next work iron and noble metal (Pt-Fe and Pd-Fe pairs) were sonophotodeposited on the self-prepared TiO_2_/zeolite Y semiconducting material [55] (Table 1, Entry 32). Different forms of deposited metals were determined by their reduction potentials. Platinum has the largest positive reduction potential among these metals. Therefore, Pt^2+^ was easily reduced to the metallic form in comparison with Fe^3+^, which is a stronger reducer and undergoes an easy oxidation during the air calcination step. As a result, mainly Pt° and Fe^3+^ forms were obtained in the Pt-Fe/TiO_2_/zeolite Y photocatalyst. In the case of the Pd-Fe/TiO_2_/zeolite Y photocatalyst Pd^2+^ form dominated over the Pd° and also the Fe^3+^ form of iron was detected. Palladium has smaller positive reduction potential than platinum therefore it could be more easily oxidized during the air calcination. A very good dispersion of platinum nanoparticles with the average size between 2 and 3 nm was obtained using the sonophotodeposition method. These good morphological properties as well as the formation of Schottky barrier between Pt° and TiO_2_ resulted in better activity of this photocatalyst in liquid phase phenol degradation. The authors also observed that the particles of deposited metals are in close vicinity to TiO_2_ particles, however formation of any specific structure (e.g., alloys, core-shell) was not confirmed by other techniques. A different reaction setup, consisted of an ultrasonic horn and a sun-imitating Xenon lamp (see Figure 12b) was checked in the sonophotodeposition of iron on the self-prepared TiO_2_/zeolite Y material [56] (Table 1, Entry 33). Iron was sonophotodeposited as the Fe^3+^ form and was mostly located in the bulk of the photocatalyst. In contrast, Fe^3+^ iron surface enrichment was observed when an ultrasound-assisted wet impregnation method was applied for the synthesis of similar Fe/TiO_2_/zeolite Y system. The authors were looking for the explanation of this behavior in different ultrasound-operating zones, from inside in the reactor sonication horn applied in the sonophotodeposition method, and from outside of the reactor in the ultrasonic bath used in the ultrasound-assisted wet impregnation method. The amount of iron on the surface of the photocatalyst was directly correlated with the results of selective photo-oxidation of benzyl alcohol into benzaldehyde, which were better for the photocatalyst prepared by the sonophotodeposition method. A simplified scheme reflecting the key of sonophotodeposition is presented in Figure 13.

## 4. Final Remarks

**Figure 14:**
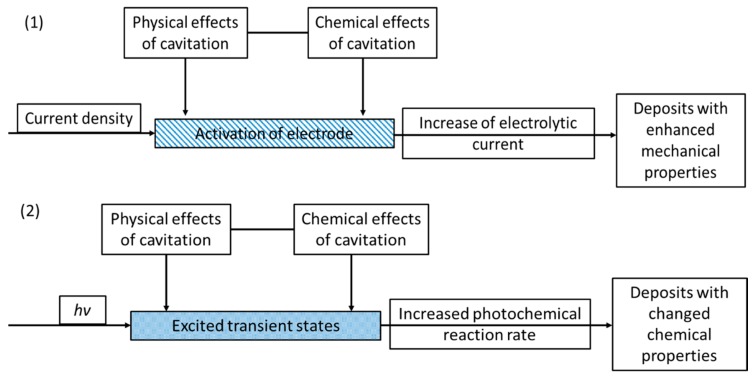
Schematic representation summarizing both (1) sonoelectrodeposition and (2) sonophotodeposition processes.

A comparison between the mechanism of sonoelectro- and sonophotodeposition is schematically presented in Figure 14. The chemistry of these processes is totally different (electrochemical reactions vs. photoexcitation), however their synergism with ultrasound is mainly based on the cavitation process and within that the enhanced mass transfer. In the case of sonoelectrodeposition the enhanced mass transfer is usually seen as the increase of electrochemical current or as the increased current pulses, depending on the type of physical effect. Such activation of electrodes results mainly in better mechanical properties of the deposits and electrocatalytic performance. On the other hand, in sonophotodeposition it influences mainly the transfer of photoexcited states. This is mostly reflected in the enhanced photochemical reaction rates, but can also end in a modified reaction pathway.

The variety of the materials obtained by sonolectrodeposition and sonophotodeposition methods is collected in Table 1. The cited examples prove that the combination of sonication with electrodeposition has already brought a new strategy for the synthesis of nanomaterials. On the other hand, not many examples of combination between sonication and photodeposition leave a free space for future research in this area. As has been mentioned in the introductory part, this short review covers a very specific topic and discusses the synergy of “sonophoto and sonoelectro” only in the field of the synthesis of materials. However, there are different applications of such synergy described in the literature. For instance, the combination of ultrasound and light in homogeneous and heterogeneous liquid systems, the influence of ultrasound on photocatalytic decomposition reactions or excitation of solutes by sonoluminescence can be found in the works of Rosenthal et al. [6], Toma et al. [24] or Gogate [57]. A wide range of application of sonoelectrochemistry was reviewed by Garcia et al. [7] and references therein. For instance, sonoelectroanalysis [20,58,59], sonoelectrochemical environmental remediation [57,60], or sonoelectrochemistry in the synthesis of nanomaterials [61] are currently very active research areas.

## Figures and Tables

**Table 1:** Examples of different materials prepared by sonoelectrodeposition and sonophotodeposition methods.

Entry	Type of Material	Synthesis Setup	Characteristics of the Material	Application/Test Reaction	Ref.
**Sonoelectrodeposition**					
1.	PbO_2_/glassy carbon electrode PbO_2_/Pt electrode	Ultrasonic bath (30 kHz, 100 W), glassy carbon rod and polyoriented platinum as the working electrodes	The activation of glassy carbon electrode in contrast to platinum electrode. This activation was explained by the formation of surface functional groups, because no change in topography of the electrode surface was seen	Potential application of PbO_2_ as an anode in: electrochemical degradation, synthesis, batteries, sensors, but not tested by authors	[16]
2.	PbO_2_/glassy carbon electrode	Sonoreactor (20 kHz, 100 W), glassy carbon rod as the working electrode, platinum wire as the counter electrode, calomel electrode as the reference	The influence of ultrasound frequency on the kinetics of electrodeposition process of lead dioxide was registered. The explanation of this phenomenon was found in the increase of OH^•^ generation	Potential application of PbO_2_ as an anode in: electrochemical degradation, synthesis, batteries, sensors, but not tested by authors	[17,18]
3.	PbO_2_/glassy carbon electrode PbO_2_/Tielectrode	Ultrasonic horn (20 kHz), or ultrasonic bath (40 kHz, 100 W), plates of graphite or titanium as the working electrodes, copper as the counter electrode	Homogeneous, free from stress and nodules. PbO_2_ film was obtained on both electrodes. A strong improvement in the quality of these deposits, a lower corrosion rate in the accelerated test reactions	Sonoelectrochemical degradation of perchloroethylene (PCE) in sulfate media; electrochemical recovery of zinc	[25]
4.	Pt/glassy carbon electrode (GC) Pt/gas diffusion layer (GDL)	Ultrasonic probe (20 kHz), glass carbon (or carbon gas diffusion layer) as the working electrode	The electrodeposition of Pt was facilitated by a decrease in nucleation overpotentials. Formation of larger nanoparticles and their agglomerates were observed	Potentially as electrodes for PEMFCs and DMFCs, but not tested by the authors	[26]
5.	Platinum nanoflowers	Ultrasonic bath (45 W), Pt working electrode, glassy carbon counter electrode, Ag reference electrode, constant potential 50 mV	Platinum layer built of irregular and fragmentized nanoparticles, gathered in self-affined larger structures, finally forming hierarchical nanoflowers	Non-enzymatic sensor of hydrogen peroxide	[28]
6.	Nanoplatinum fractal structures	Ultrasonic bath, Pt/Ir electrodes as the working electrode, Pt wire as the counter electrode, constant overpotential 10 V	The nanoplatinum displayed: fractal features, homogeneous size distribution and very high electroactive surface. Formation of stable nanostructures were promoted by a high duty cycle (900 mHz) and reduction of amorphous structure due to cavitation effect	Non-enzymatic and enzymatic sensors for measuring hydrogen peroxide and glucose	[29]
7.	CaP/carbon fibers	Ultrasonic bath (40 kHz, 2.16 W/cm^2^), carbon fabric as the working electrode, constant current density (20 mA/cm^2^)	Better morphology: a uniform coating with small crystals and good adhesive strength, was obtained under this condition in comparison with a silent mode	Biomedical application: bioceramic composites used for the reconstruction of bone defects	[30]
8.	CaP/carbon fibers	Ultrasonic bath (40 kHz, 2.16 W/cm^2^), carbon fabric as the working electrode, platinum plate as the counter electrode, different current densities: 5, 8, 13, 20, and 34 mA/cm^2^	Different morphology of the CaP coatings depends on the current density. More uniform structures with smaller crystal sizes were obtained at higher values of current density	Biomedical application	[31]
9.	Hydroxyapatite (HA)/porous carbon composite scaffolds	Ultrasonic stirring, porous carbon, constant voltage 3 V	A homogeneous coating with HA crystals across external as well as internal surfaces of the porous carbon scaffold was obtained	Biomedical application: in vitro 3D culture of osteoblasts	[32]
10.	CaP-coated C/C composites	Ultrasonic device (25 kHz, 100 W), C/C electrode as the cathode, graphite as the anode, different voltages: 2.0, 2.4, 2.7, and 3.0 V, controlled temp. 50 ± 3 °C	The most homogeneous coating was obtained at 2.4 V and formed an interlocking structure along the depth direction of the coating without any defects or uncovered areas. This resulted in improved adhesive and cohesive strength of the coating	Biomedical application, especially as dental and medical implant materials	[33]
11.	CdS/TiO_2_NT	Ultrasonic bath (40 kHz, 2.4 kW/m^2^), TiO_2_ NT as the working electrode, Pt foil as the counter electrode, constant current density (5 mA/cm^2^), constant temperature 50 °C	TiO_2_ nanotubes were successfully filled with CdS small-sized nanoparticles with more homogeneous distribution. A stronger photocurrent and extended photoresponse to the visible light were observed for such composites	Potential application in photocatalytic reactions, but not performed by the authors	[34]
12.	Cu/Graphite	Ultrasonic probe (20 kHz, 20% output), graphite electrode as the cathode, Pt electrode as the anode, Ag electrode as the reference electrode, different set of temperatures: 25, 20, 15, 10, and 5 °C	A maximum value of compressive residual stress in the Cu films was registered at 5 °C. This result has a direct influence on the mechanical properties of the film, as the maximum hardness and elasticity occurred also at the lowest deposition temperature	Potential application in electronic industry, but not tested by the authors	[35]
13.	Cu/Graphite	Ultrasonic bath (30 kHz, 60 W); graphite electrode as the cathode, copper electrode as the anode, calomel electrode as the reference; temperatures selected for this experiment were as following: 25, 19.5, −1 and −3 °C	Cleaner nanorange deposits of copper were obtained under sonication. Different morphology of Cu films was registered at different deposition temperatures: from inhomogeneously deposited distorted grains at 25 °C to uniform coatings with fine grains at −3 °C	Potential application in electronic industry, but not tested by the authors	[36]
14.	Poly(pyrrole)/Prussian blue (PB) nanocomposite	Ultrasonic horn (20 kHz, 130 W) with different amplitudes: 20%, 40%, and 60%; ITO electrodes	With the increasing ultrasound amplitude the morphology of the film changed from large aggregates to small particles homogeneously distributed over the electrode surface	Electrocatalytic reduction of H_2_O_2_. Potential application in electrocatalysis (sensors/biosensors)	[37]
15.	Poly(pyrrole)/dodecylbenzenesulphonate (DBS) film	Ultrasonic horn (20 kHz, 130 W) with different amplitudes: 20%, 40%, and 60%; platinum electrode	Different morphology of the films obtained under silent and sonochemical conditions reflected in a distinct voltammetric behavior of electrodes. Diminished the charge-transfer resistance of the films	Potential application as electrochemical based devices: sensors, biosensors and supercapacitors	[38]
16.	Co-Pt NPs	Pulse mode operating ultrasonic horn acted as the cathode and ultrasound emitter, platinum plate as the counter electrode	An ordering of Co-Pt phase, hard magnetic properties and formation of metallic core-carbon onion shell structure after sonoelectrodeposition with annealing in CO atmosphere	Potential magnetic application (e.g., ultrahigh-density magnetic storage media), but not tested by the authors	[39]
17.	FePt NPs	Pulse mode operating ultrasonic horn acted as the cathode and ultrasound emitter, platinum plate as the counter electrode	Improvement of magnetic properties and ordering of crystal structure of FePt were obtained when sonoelectrodeposition was followed by annealing at high temp	Potential magnetic application (e.g., ultrahigh-density magnetic storage media), but not tested by the authors	[41]
18.	FePd NPs	Pulse mode operating ultrasonic horn acted as the cathode and ultrasound emitter, platinum plate as the counter electrode	Sonoelectrodeposition with the following annealing at high temp. resulted in formation of the ordered L1_0_ crystal structure and hard magnetic properties of FePd NPs	Potential magnetic application (e.g., ultrahigh-density magnetic storage media), but not tested by the authors	[40]
19.	Ni-Co/Al_2_O_3_	Ultrasonic power (0–160 W), nickel plate as anode, polished mild steel sheet as cathode, under pulse reverse current	Uniform, compact coating with a fine grains and enhanced mechanical properties	Potential application in automobile/aerospace industry, but not tested by the authors	[42]
20.	Zn-Ni-Al_2_O_3_	Ultrasonic horn (20 kHz, 150 W) with ultrasonic power applied 0.7 W/cm^2^, Zn plate as the anode, Cu as the cathode	Uniform dispersion of nano-alumina in the Zn-Ni matrix. Different composition of the composite layers: the outermost layer consists of Al_2_O_3_ and Zn(OH)_2_ while transitional layer contains Al_2_O_3_, ZnO, Zn, and Ni	Potential application as anti-corrosion coatings	[43]
21.	Zn-Ni-Al_2_O_3_	Ultrasonic horn (20 kHz, 150 W) different ultrasonic power applied (from 0 W/cm^2^ to 1.2 W/cm^2^), Zn plate as the anode	Increased content and more uniform dispersion of nano-alumina particles resulted in improved anticorrosion property and hardness of the composite coating (0.7 W/cm^2^)	Potential application as anti-corrosion coatings	[44]
22.	Ag NPs	Ultrasonic bath (20 kHz, 100 W), stainless steel as a the cathode, Ru-Ti alloy as a the counter electrode, controlled-current 60 mA	Shaped silver NPs were obtained: spheres with a diameter about 30 nm, wires with a diameter 30 nm and length 200–900 nm and dendrites, formed with increasing concentration of silver solution	Potential application in microelectronics, optical, electronics, magnetic devices, but not tested by the authors	[45]
23.	Ag NPs loaded on active carbon	Sonicator working in a pulse mode, silver plate used as the cathode, platinum plate as the anode	Ag NPs with the size of 4–30 nm dispersed in a non-toxic solution due to the use of silver plate as a source of silver ions. In the next step Ag NPs were loaded on the surface of active carbon	Antibacterial activity against *Escherichia coli*	[46]
24.	Au nanorods deposited on gold surface (Au-Au NR)	Ultrasonic bath (45 W) and working Au electrode	Special size, shape and structure of nanorods were obtained: a width of 80–120 nm, a length of 140–370 nm, and an aspect ratio of 1.6–3.5	Nitrofurazone sensor	[47]
25.	Cu-Ag NPs	Ultrasonic horn with titanium probe used as the working electrode working in a pulse mode; copper rod as the counter electrode, saturated mercury sulfate as the reference electrode	Cu core-Ag shell structure (7 nm diameter of NPs) obtained by combination of sonoelectrodeposition for the inner core and galvanic replacement reaction for outer shell	Bactericidal properties against *Staphylococus* aureus and *Escherichia coli* bacteria	[48]
26.	Fe-Cr alloy NPs	Ultrasonic horn with titanium probe used as the working electrode working in a pulse mode (20 kHz); platinum net used as the counter electrode	Process efficiency decreases with increasing temperature of the electrolyte. Structural and morphological features of the NPs are not influenced by the synthesis temperature. The crystalline structure of NPs depends on the electrolyte’s composition	Potential technological application (good mechanical, anti-corrosive and water-resistant properties), but not tested by the authors	[49]
27.	Iridium NPs on a copper plate	Ultrasonic homogenizer (20 kHz), copper plate as the cathode, platinum plate as the anode	Reduced defects, including cracks in the iridium deposits. Accelerated rate of iridium deposition	Potential industrial and chemical application (as an inert material), but not tested by the authors	[50]
28.	NiZnS alloy	Ultrasound irradiation of 20 kHz at different current densities, pulse electrodeposition at various duty cycles at 10 Hz; nickel mesh electrode	The smallest size of deposit particles (17 nm), uniform coatings in fine-grained structures of the alloy, better surface morphology, the highest surface area, the highest exchange current density (8.25 × 10^−3^ A/cm^2^) and high current density (0.42 A/cm^2^) were achieved in pulse sonoelectrodeposited electrodes	Oxygen evolution reaction (OER) in alkaline media	[51]
**Sonophotodeposition**					
29.	Pd/TiO_2_P90	Ultrasonic bath and 6 W Hg lamp (λ_max_ = 254 nm)	Total reduction of palladium, despite the air calcination step. In contrast, surface PdO forms were detected in the material prepared by pure photodeposition. This confirmed the role of sonication in the reduction process	Gas phase photocatalytic degradation of methanol	[52]
30.	Pd-Au/TiO_2_P90	Ultrasonic bath and 6 W Hg lamp (λ_max_ = 254 nm)	Formation of random alloys between Pd-Au NPs and SMSI effect between NPs and TiO_2_ for one specified composition of metals: 1 wt% Pd50-Au50/TiO_2_ P90	Gas phase selective photocatalytic oxidation of methanol to methyl formate.	[53]
31.	Pd-Cu/TiO_2_P90	Ultrasonic bath and 6 W Hg lamp (λ_max_ = 254 nm)	SMSI effect between Pd-Cu NPs and TiO_2_ for one specified composition of metals: 1 wt% Pd-Cu(1-1)/TiO_2_ P90. Retarding effect of Cu on total reduction of Pd	Gas phase selective photocatalytic oxidation of methanol to methyl formate	[54]
32.	Pd-Fe/TiO_2_/Zeolite YPt-Fe/TiO_2_/Zeolite Y	Ultrasonic bath and 6 W Hg lamp (λ_max_ = 254 nm)	Mainly Pt° and Fe^3+^ in Pt-Fe/TiO_2_/Ze and Pd^2+^ and Fe^3+^ in Pd-Fe/TiO_2_/Ze were formed (reduction potentials dependency). A very good dispersion and control over the particle size in the case of Pt nanoparticles	Liquid-phase photocatalytic oxidation of phenol under UV lamp	[55]
33.	Fe/TiO_2_/Zeolite Y	Ultrasonic horn (20 kHz, 700 W, 25% of amplitude) and sun-imitating Xenon lamp (240–2000 nm)	4–5 nm sized Fe^3+^ NPs mainly located in the bulk of the material due to the physical effect of ultrasound	Liquid-phase selective photocatalytic oxidation of benzyl alcohol into benzaldehyde in acetonitrile under UV-Vis irradiation	[56]
